# Research trends in endoscopic applications in early gastric cancer: A bibliometric analysis of studies published from 2012 to 2022

**DOI:** 10.3389/fonc.2023.1124498

**Published:** 2023-04-11

**Authors:** Yuan Liu, Haolang Wen, Qiao Wang, Shiyu Du

**Affiliations:** ^1^ Graduate School of Beijing University of Chinese Medicine, Beijing, China; ^2^ Department of Gastroenterology, China-Japan Friendship Hospital, Beijing, China

**Keywords:** early gastric cancer (EGC), endoscopy, bibliometric analysis (BA), CiteSpace, VOSviewer

## Abstract

**Background:**

Endoscopy is the optimal method of diagnosing and treating early gastric cancer (EGC), and it is therefore important to keep up with the rapid development of endoscopic applications in EGC. This study utilized bibliometric analysis to describe the development, current research progress, hotspots, and emerging trends in this field.

**Methods:**

We retrieved publications about endoscopic applications in EGC from 2012 to 2022 from Web of Science™ (Clarivate™, Philadelphia, PA, USA) Core Collection (WoSCC). We mainly used CiteSpace (version 6.1.R3) and VOSviewer (version 1.6.18) to perform the collaboration network analysis, co-cited analysis, co-occurrence analysis, cluster analysis, and burst detection.

**Results:**

A total of 1,333 publications were included. Overall, both the number of publications and the average number of citations per document per year increased annually. Among the 52 countries/regions that were included, Japan contributed the most in terms of publications, citations, and H-index, followed by the Republic of Korea and China. The National Cancer Center, based in both Japan and the Republic of Korea, ranked first among institutions in terms of number of publications, citation impact, and the average number of citations. Yong Chan Lee was the most productive author, and Ichiro Oda had the highest citation impact. In terms of cited authors, Gotoda Takuji had both the highest citation impact and the highest centrality. Among journals, *Surgical Endoscopy and Other Interventional Techniques* had the most publications, and *Gastric Cancer* had the highest citation impact and H-index. Among all publications and cited references, a paper by Smyth E C et al., followed by one by Gotoda T et al., had the highest citation impact. Using keywords co-occurrence and cluster analysis, 1,652 author keywords were categorized into 26 clusters, and we then divided the clusters into six groups. The largest and newest clusters were endoscopic submucosal dissection and artificial intelligence (AI), respectively.

**Conclusions:**

Over the last decade, research into endoscopic applications in EGC has gradually increased. Japan and the Republic of Korea have contributed the most, but research in this field in China, from an initially low base, is developing at a striking speed. However, a lack of collaboration among countries, institutions, and authors, is common, and this should be addressed in future. The main focus of research in this field (i.e., the largest cluster) is endoscopic submucosal dissection, and the topic at the frontier (i.e., the newest cluster) is AI. Future research should focus on the application of AI in endoscopy, and its implications for the clinical diagnosis and treatment of EGC.

## Introduction

Gastric cancer is the fifth most common cancer and the third leading cause of cancer-related deaths worldwide, accounting for approximately 1,000,000 new cases in 2018 and an estimated 783,000 deaths (1). Early gastric cancer (EGC) refers to cancer tissue that is restricted to the gastric mucosa or submucosa, regardless of lymph node metastases. A retrospective analysis of over 100,000 patients from the Japan Gastric Cancer Association reveals that the 5-year disease-specific survival rate for EGC is 98.2%, whereas the 5-year disease-specific survival rate for other kinds of gastric cancer ranges from 29.2% to 77.6% (2). Therefore, early diagnosis and treatment are essential for improved EGC patient outcomes (3).

Gastric cancer is diagnosed by endoscopic examination, which locates the tumor in the stomach, and by biopsies, which are histopathologically examined to determine its macroscopic type (4). EGC causes very few symptoms (5) and is therefore mainly diagnosed through endoscopy (6). Compared with other screening approaches, endoscopy has played an increasingly vital role in the diagnosis of stomach cancer (7). In the field of gastric disease, the endoscopic techniques commonly used are white light endoscopy (WLE), chromoendoscopy, magnifying endoscopy–narrow-band imaging (ME-NBI), blue laser imaging (BLI), and endoscopic ultrasonography (EUS) (8). Each type has unique advantages in the diagnosis of EGC. Endoscopic resection (ER) is the optimal treatment for early gastric cancer, provided that substantial risk factors for lymph node metastases are not present (4). There are two ER techniques: endoscopic submucosal dissection (ESD) and endoscopic mucosal resection (EMR). ESD involves the dissection of the submucosa beneath the lesion after resecting the mucosa around the lesion with high-frequency diathermy, and EMR involves the resection of the elevated lesion with high-frequency diathermy (9). Compared with EMR (4), ESD takes practitioners longer to master and has a longer procedure time, and an almost three times higher perforation rate. However, curative resection rates, en bloc resection rates, and complete resection rates are higher, and local recurrence rates are lower (10).

Endoscopic applications in EGC is developing rapidly, so it is essential to stay abreast of changes in the related body of literature. Bibliometric analysis (BA) can be applied to summarize the trends and hotspots of global research and has been widely applied in many fields (11). BA utilizes public literature databases, employing mathematical and statistical methods to quantitatively analyze the literature data and its metrological characteristics, visualizing the research frontiers and hotspots. In addition, it can accurately indicate what the most influential research is, as well as theoretical foundations for future research, thus supporting decision-makers (12–14). However, no BA research summarizing the literature on the application of endoscopy in EGC has been published.

Therefore, by identifying and collecting literature data from databases, we can determine the countries, institutions, authors, and journals with the highest citation impacts, or the greatest number of publications pertaining to endoscopic applications in EGC. Using BA, this study aims to describe the development of endoscopic applications in EGC from 2012 to 2022, as well as the current research progress, hotspots, and emerging trends in endoscopic applications in EGC, thereby improving other researchers’ understanding of future research trends and hotspots in endoscopic applications.

## Method

### Database

The data source we used was the Science Citation Index Expanded^®^ (SCIE) of Clarivate™’s (Philadelphia, PA, USA) Web of Science™ Core Collection (WoSCC). The WoSCC is the most utilized data source in bibliometric analysis because of it publishes only articles that meet its stringent evaluation criteria. As a journal citation sub-database of WoSCC, SCIE is a multidisciplinary comprehensive database of publications in the field of natural science, with over 8,600 authoritative global journals covering 176 topic categories.

### Search strategy and screening criteria

**Figure 1:**
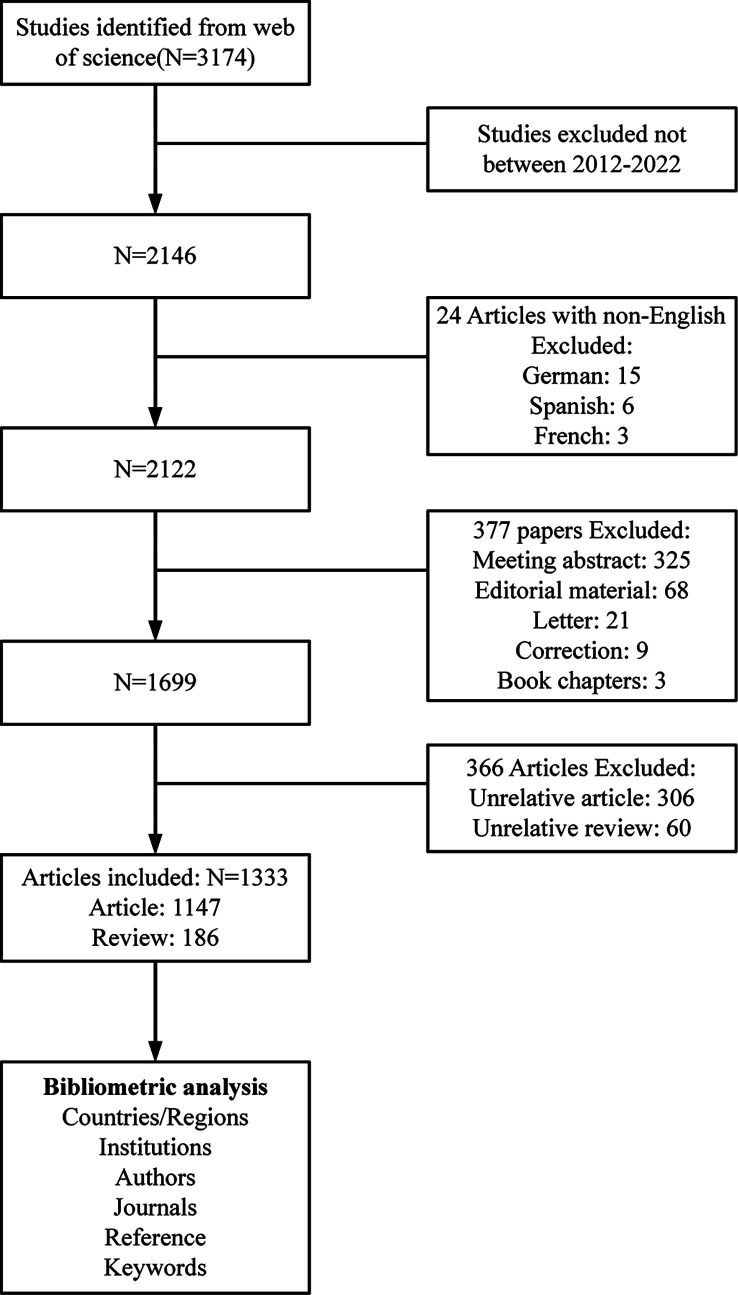
Flowchart of including and excluding publications, performed by Microsoft Visio.

We searched the databases for publications pertaining to endoscopic applications in the field of EGC. Two researchers from our organization simultaneously analyzed the information on each paper, and the search was completed within 1 day to ensure that no data modifications occurred. The publications’ titles, keywords, abstracts, authors, institutions, and reference records were downloaded and saved in plain text format. The search formula was [TS= (“early gastric neoplasm” OR “early gastric cancer” OR “early gastric carcinoma” OR “early gastric tumor” OR “early-stage gastric cancer” OR “early-stage gastric neoplasm” OR “early-stage gastric carcinoma” OR “early-stage gastric tumor”)) AND TS= (“endoscopy” OR “endoscopic” OR “endomicroscopy” OR “White light endoscopy” OR “Chromoendoscopy” OR “Narrow band imaging” OR “Blue laser imaging” OR “Magnifying endoscopy” OR “Confocal laser endomicroscopy” OR “Endocytoscopy” OR “Endoscopic ultrasonography”]. The screening criteria are shown in **Figure 1**. After the initial search, we selected two authors (YL and HLW) to review and screen the searched publications, independently, based on the following inclusion criteria: (1) the language of the publication was English; (2) the publication type was either article or review; (3) the article was published between 31 October 2012 and 31 October 2022; and (4) the publication related to a study of both EGC and endoscopy. A study was considered to concern EGC if it involved EGC patients (either preoperative or postoperative EGC), animal models of EGC, or cell models of EGC, and to concern endoscopy if it involved any form of endoscopy. Disputes between the two authors were settled through consultation. A total of 1,333 articles were found to meet the inclusion criteria and were included in the final analysis.

### Data analysis and visualization

Two writers downloaded and evaluated the data independently to ensure data accuracy and research reproducibility. Microsoft Excel^®^ 2019MSO (version 2210 Build 16.0.15726.20070; Microsoft Corporation, Redmond, WA, USA), GraphPad Prism (version 8.0.1.244; GraphPad Software Inc., CA, USA), Charticulator (version 2.2.0), and R (version 4.1.3; The R Foundation for Statistical Computing, Vienna, Austria) were used to analyze files and export flow charts, histograms, line charts, chord diagrams, and tables of the top-cited or most productive authors, and of the countries/regions, publications, journals, and institutions making the greatest contribution to the field. The H-index is a hybrid index established by Hirsch for evaluating academic achievements (15). We also applied Bradford’s law of scattering to describe and find the “core journals”, which can be defined as follows (16): “If journals are arranged in order of decreasing productivity of publications, they can be divided into a nucleus of periodicals, more particularly devoted to the subject and several groups or zones containing the same articles as the nucleus, with the number of journals in each part proportional to 1 : n :n².” Price’s law is also extensively employed in BA and states that the square root of the total number of writers in a certain field contributes to 50% of the publications on a given topic (17).

In this study, we mainly used CiteSpace (version 6.1.R3) and VOSviewer (version 1.6.18), which are the two best information visualization software packages for data visualization in the field of knowledge graphs available worldwide. CiteSpace is a Java-based application developed by Professor Chaomei Chen (18). Its major advantage is that it enables cluster analysis, timeline view, and citation burst detection. VOSviewer was developed by Eck and Waltman (19) and is useful for co-occurrence analysis and co-citation analysis. Combining the two software packages can enable the depiction of the progression, coordination, structure, and other aspects of knowledge fields by constructing linkages and visually analyzing literary knowledge items (20). If one study is generated in collaboration with several countries, institutions, and authors, then each country, institution, and author is counted as a contributor, which is the basis for the institutional, national, and author cooperation networks.

The CiteSpace parameters were set as follows:

(1) For the cited author, cited institution, and cited reference analyses, the time slicing function, in 1-year slices, was first used to filter articles published between 2012 and 2022. Subsequently, all options in the term source were selected, the node types were selected one at a time, the selection criterion was TOP50 (select the top 50 most cited or most frequently occurring items in each time slice), and pruning was conducted by way of the Pathfinder function on the sliced networks.
(2) For keywords analysis, the time slicing function, in 1-year slices, was again first used to filter articles published between 2012 and 2022. Subsequently, all options in the term source except Keywords Plus were selected, the node type selected was Keyword, the selection criterion was TOP30, and pruning was conducted by way of the Pathfinder function on the merged networks. Each node in the figure indicated an observation: institution, cited author, co-cited literature, or keyword.

Prior to conducting analysis *via* CiteSpace, we combined alternative versions of the same institution, author, or keyword For instance, “Chinese Academy of Medical Sciences” was combined with “Chinese Academy of Medical Sciences and Peking Union Medical College”, “Mi-Young Kim” was combined with “M Y Kim”, and “long-term outcome” was combined with “longterm outcome”. We then merged the subordinate units of the same institution, such as “Yokohama City University” and “Yokohama City University Medical Center,” and excluded meaningless keywords such as “gastric cancer”. The manipulations mentioned above were key for better visualization and more accurate analysis.

### Research ethics

The data used in our study were available from an open-source database, and, therefore, their use does not require ethics approval.

## Result

### Global trends of publication and citation

**Figure 2:**
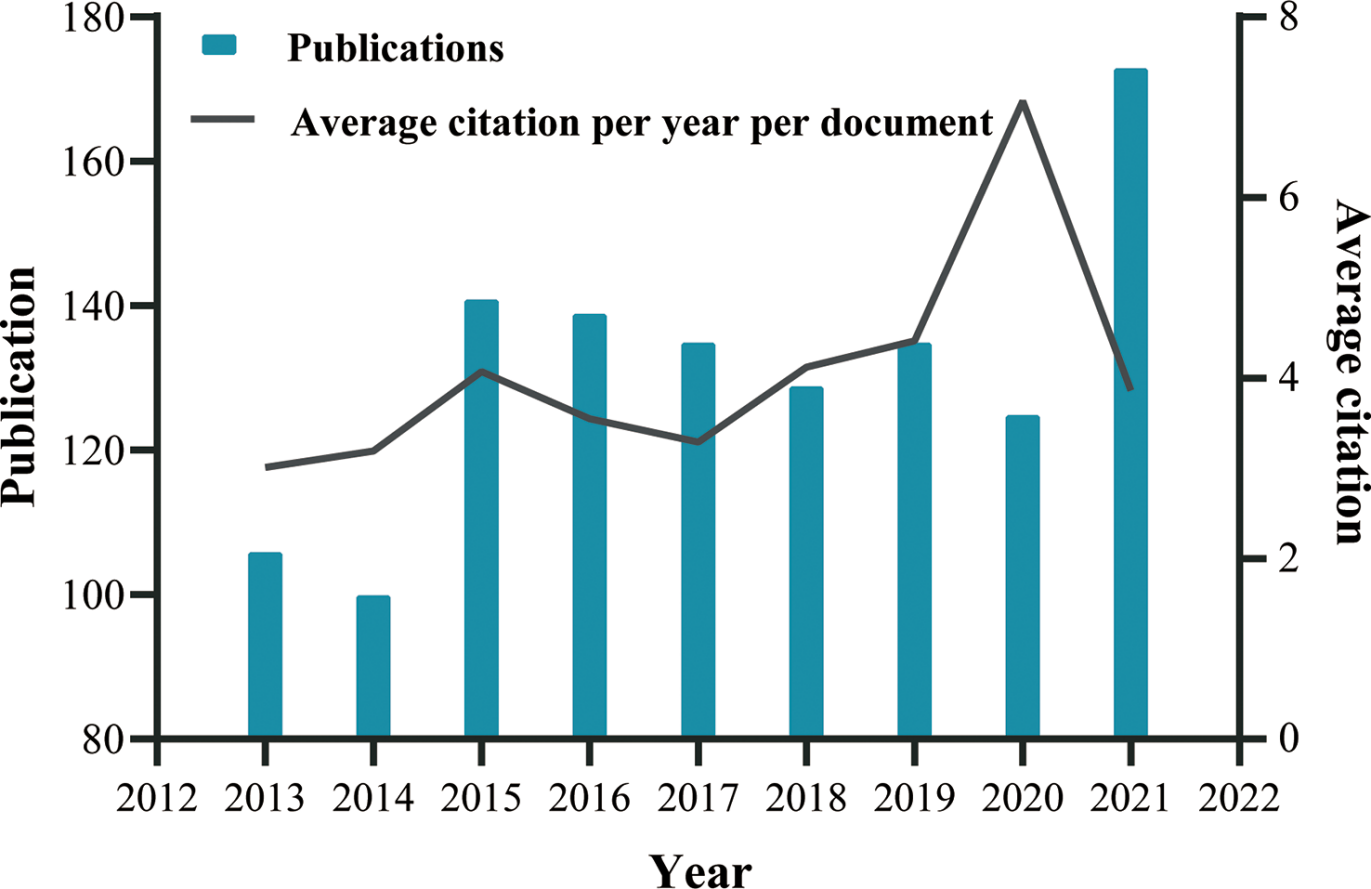
Global trend of publications and average citation per year per document on endoscopic application in ECG from 2013 to October 2021, performed by GraphPad Prism 8. For more accurate visualization, we omit the incomplete data for 2012 and 2022.

Based on the data searching formula, we retrieved a total of 1,333 publications from the SCIE sub-database of WoSCC, comprising 1,147 articles and 186 reviews (**Figure 1**). It was clear from our analysis that the number of publications significantly increased in 2015 and 2021, and was steady, with some fluctuation, in other years (**Figure 2**). Altogether, these 1,333 publications were cited 21,443 times, with each publication being cited an average of 16.09 times. **Figure 2** also illustrates the average number of citations per year per document, which has continuously increased. These phenomena indicate that research on the application of endoscopy in EGC is receiving more and more attention.

### Contributions of the most productive countries/regions

**Figure 3:**
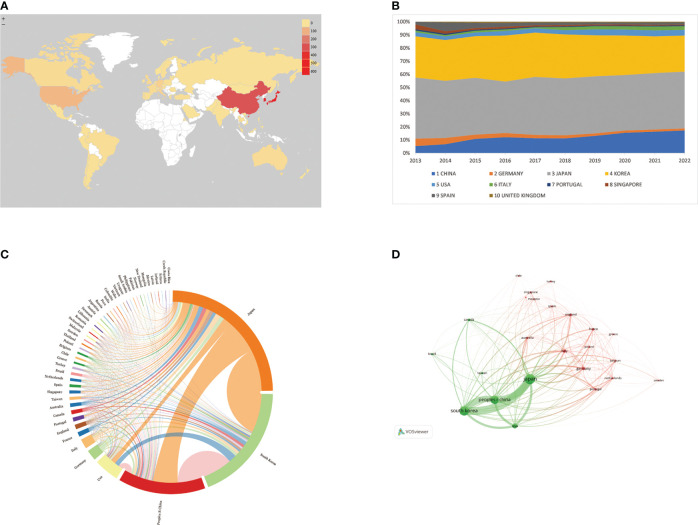
**(A)** World map based on the total publications of different countries/regions, performed by Microsoft Excel. **(B)** The changing consistency of the annual publication quantity in the top 10 countries/regions from 2013 to 2022, performed by Microsoft Excel. **(C)** Charticulator's map of worldwide countries/regions. The thickness of links between countries regions symbolizes collaboration, whereas ring surface area denotes publications. **(D)** VOSviewer's map showing countries/regions with more than 4 publications. Size of nodes and thickness of linkages indicate publishing quantity and connection strength.

A total of 52 countries/regions have published relevant studies. As shown in the world map in **Figure 3A**, the countries that produced more than 200 studies were Japan, the Republic of Korea, and the People’s Republic of China. The annual publication trends of the top 10 nations, shown in **Figure 3B**, reveal that Japan and the Republic of Korea have consistently published the largest numbers of studies, and that, in the last 10 years, the number produced by the People’s Republic of China has shown an increasing tendency. **Table 1** provides details of the top 10 productive countries, and shows that Japan ranked first, with 504 articles. In addition, Japan ranked first in terms of the number of citations (9,333), followed by the Republic of Korea (5,782) and the People’s Republic of China (2,723). Japan also ranked first in terms of the H-index, and these findings reflect that Asian nations are positioned more favorably in these fields. However, although researchers in European countries have not published as many articles as those in Asian countries, the average number of citations per paper for articles published by the former was much higher (over 39), indicating the high quality of their research. As seen in **Figure 3C**, the cooperative links among Japan, the Republic of Korea, the People’s Republic of China, and the USA were robust, whereas cooperation among the other nations was limited. **Figure 3D** illustrates the collaboration of the countries/regions with more than four publications. A total of 24 countries/regions are included in this co-authorship visualization map; the links between nodes represent the collaboration between countries/regions. The thicker the links, the closer the degree of collaboration [this is referred to as total link strength (TLS)]. The top five countries in terms of TLS were Japan, the Republic of Korea, the People’s Republic of China, the USA, and Italy.

**Table 1:** Top 10 productive countries/regions related to the endoscopic application in early gastric cancer.

Rank	Countries /regions	TLS	Documents	Percentage of documents	Citations	Average publishing year	Average citations	H-index
1	Japan	3506	504	37.81%	9333	2017.44	18.52	91
2	South Korea	2849	386	28.96%	5782	2017.23	14.98	51
3	China	2620	286	21.46%	2723	2018.67	9.52	43
4	USA	1047	82	6.15%	1499	2017.84	18.28	28
5	Germany	576	35	2.63%	1911	2016.51	54.6	20
6	Italy	643	32	2.40%	1279	2018.72	39.97	17
7	England	437	15	1.13%	1966	2018.33	131.07	16
8	France	350	15	1.13%	1112	2016.33	74.13	12
9	Portugal	407	13	0.98%	823	2018.77	63.31	13
10	Canada	337	13	0.98%	237	2018.69	18.23	11

### Contributions of top journals

**Figure 4:**
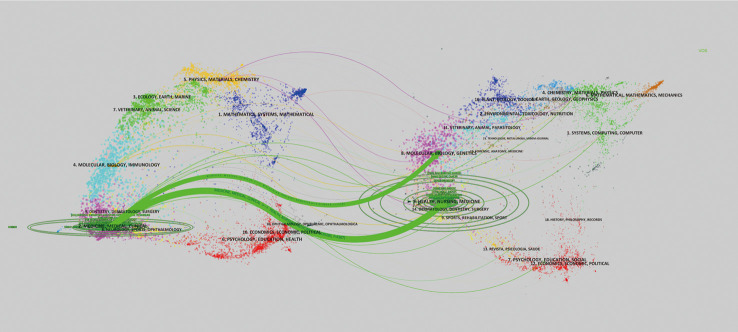
The left side of this dual map is the citing paper map, while the right side is the cited paper map. The curve represents the citation line. On the left side, the horizontal and vertical ellipse axes reflect publications and authors in corresponding journals.

The publications appeared in a total of 226 journals, of which 15 journals could be defined “core journals” according to the aforementioned Bradford’s law of scattering (**Table 2**). The top three most productive journals were *Surgical Endoscopy and Other Interventional Techniques* (131, 9.83%), *Gastric Cancer* (100, 7.50%), and *World Journal of Gastroenterology* (79, 5.93%). Furthermore, the total number of citations and H-index for the journal *Gastric Cancer* were 2,475 and 29, respectively, which were the highest among all journals; however, *Endoscopic* had the largest average number of citations (50.32). According to the 2021 Journal Citation Report (JCR), five of the core journals were in Q1; among these, *Endoscopic* ranked first, with an impact factor (IF) of 10.437. **Figure 4** is a dual map of the journals and shows the link between citing and cited journals. Two main citation paths were identified: (1) medicine, medical, and clinical—health, nursing, and medicine (z = 6.1539283, f = 4,367); and (2) medicine, medical, and clinical—molecular, biology, and genetics (z = 2.0139656, f = 1,538). The citing papers were mainly located in three fields: (1) medicine, medical, and clinical; (2) molecular, biology, and immunology; and (3) dentistry, dermatology, and surgery. The cited papers were mainly concentrated in three fields: (1) molecular, biology, and genetics; (2) health, nursing, and medicine; and (3) dermatology, dentistry, and surgery.

**Table 2:** The top 15 productive journals related to endoscopic application in early gastric cancer.

Rank	Journal	Countries	Documents	TLS	IF (2021)	JCR (2021)	Total citations	average citations	H-index
1	Surgical Endoscopy and Other Interventional Techniques	USA	131	1121	3.453	Q2	1968	15.02	25
2	Gastric Cancer	Japan	100	1093	7.701	Q1	2475	24.75	29
3	World Journal of Gastroenterology	USA	79	528	5.374	Q2	1345	17.03	21
4	Gastrointestinal Endoscopy	USA	51	654	10.396	Q1	1745	34.22	25
5	Digestive Endoscopy	Japan	45	652	6.337	Q1	1593	35.40	19
6	Medicine	USA	42	202	1.817	Q3	265	6.31	10
7	Endoscopy	Germany	37	434	10.437	Q1	1862	50.32	21
8	Digestion	Switzerland	35	235	3.632	Q3	367	10.49	11
9	Journal of Gastroenterology and Hepatology	Australia	34	187	4.369	Q2	255	7.50	10
10	Gut and Liver	South Korea	29	261	4.321	Q2	437	15.07	12
11	Annals of Surgical Oncology	USA	24	229	4.339	Q1	489	20.38	13
12	Journal of Gastric Cancer	South Korea	23	134	3.197	Q3	89	3.87	6
13	Gastroenterology Research and Practice	USA	22	126	1.919	Q4	171	7.77	9
14	Plos One	USA	21	159	3.752	Q2	183	8.71	8
15	Digestive Diseases and Sciences	USA	21	156	3.487	Q3	250	11.90	11

### Analysis of institutions

**Figure 5:**
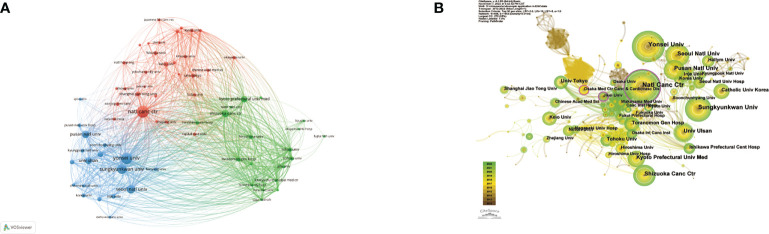
**(A)** Network visualization of the institutions with more than 10 publications. **(B)** Network visualization map of institutional collaboration analysis. Size of nodes and thickness of linkages signify publication counts and connection strength. The nodes with an outer purple ring reveal high centrality.

The 10 most prolific institutes that engaged in research regarding endoscopic applications in EGC were identified; all are located in Japan and/or the Republic of Korea (**Table 3**). The top three most prolific institutes are the National Cancer Center [NCC, which consists of NCC (Japan) and NCC (Republic of Korea)], Yonsei University, and Sungkyunkwan University, with a total of 100, 90, and 72 publications, respectively. NCC has the largest total number of citations (2,737) and the largest average number of citations (27.37). There were 67 institutions that published more than 10 studies (**Figure 5A**). The top three institutions in terms of TLS were the NCC (TLS = 2,434), the Shizuoka Cancer Center (TLS = 1,901), and Yonsei University (TLS = 1,489). Many institutions were lack of partnership with other institutions (**Figure 5B**).

**Table 3:** Top 10 institutes in the publications related to endoscopic application in early gastric cancer.

Rank	Institutions	Countries/Regions	Documents	TLS	Citations	Average citations
1	National Cancer Center	Japan, South Korea	100	2434	2737	27.37
2	Yonsei University	South Korea	90	1489	1349	14.99
3	Sungkyunkwan University	South Korea	72	1395	1075	14.93
4	Seoul National University	South Korea	59	1182	918	15.56
5	Shizuoka Cancer Center	Japan	47	1901	994	21.15
6	Pusan National University	South Korea	49	756	721	14.71
7	Kyoto Prefectural University of Medicine	Japan	40	1284	916	22.9
8	University of Ulsan	South Korea	41	798	725	17.68
9	Toranomon Hospital	Japan	26	1163	682	26.23
10	Catholic University of Korea	South Korea	33	552	370	11.21

Centrality is a reflection of a node’s importance and serves as an indicator of the key nodes in the networks. In general, a node with a centrality value of over 0.1 is a critical node that links several other nodes and is denoted by a purple outer ring (18). Although the Osaka Medical Center for Cancer and Cardiovascular Diseases and Jikei University were not among the top 10 most productive institutions, their centralities, of 0.14 and 0.13, respectively, were almost identical to that of the NCC.

### Analysis of authors and cited authors

**Figure 6:**
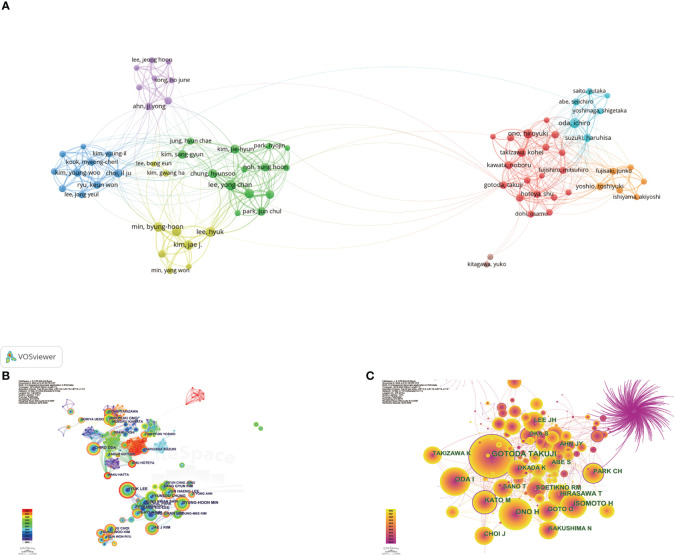
**(A)** Network visualization of the 79 "core authors" with each author had published at least 16 articles. **(B)** Network visualization map of authors collaboration analysis. Redder and thicker outer rings indicate more recent writing activity. **(C)** Network visualization map of cited authors collaboration analysis. Node size indicates citations, and high-centrality nodes have a purple ring.

The top 10 most productive authors and the co-cited authors with the highest citations are shown in **Table 4**. Yong Chan Lee, Hyuk Lee, and Ichiro Oda were the three most prolific authors, with 47, 45, and 43 publications, respectively. Ichiro Oda had the largest total number of citations, the highest average number of citations and the highest H-index (i.e., 2,048, 47.63 times per paper, and 24, respectively), followed by Jun Haeng Lee (1,158, 30.47 times per paper, and 22, respectively), and Yong Chan Lee (933, 19.85 times per paper, and 19, respectively). Based on Price’s law, 79 authors who had each published at least 16 articles were defined as “core authors” (**Figure 6A**). These authors were separated into two groups depending on their country of origin. In general, the authors’ centrality was very low (less than 0.05), reflecting the fact that many authors have no links, which indicates a general lack of cooperation (**Figure 6B**). In the co-cited visualization analysis (**Figure 6C**), Gotoda Takuji, Ono Hiroyuki, and Oda Ichiro were the top three authors, with 663, 390, and 291 citations, respectively. The centrality of both Gotoda Takuji and Park Chan Hyuk was 0.19, which was higher than the centrality of other cited authors, indicating that these two authors have a significant impact on this field of research.

**Table 4:** Top 10 most productive authors and top 10 co-cited authors with the highest citations.

Rank	Author	Countries/Regions	Documents	Total citations	H-index	Co-cited author	Countries/Regions	Centrality	Total citations	H-index
1	Lee, Yong Chan	South Korea	47	933	19	Gotoda, Takuji	Japan	0.19	663	17
2	Lee, Hyuk	South Korea	45	768	16	Ono, Hiroyuki	Japan	0.01	390	16
3	Oda, Ichiro	Japan	43	2048	24	Oda, Ichiro	Japan	0.05	291	24
4	Min, Byung-Hoon	South Korea	41	535	14	Isomoto, Hajime	Japan	0.04	252	4
5	Lee, Sang Kil	South Korea	40	775	17	Hirasawa, Toshiaki	Japan	0.03	212	11
6	Kim, Jae J	South Korea	38	636	15	Kato, Mototsugu	Japan	0.12	199	10
7	Ono, Hiroyuki	Japan	38	619	16	Ahn, Ji Yong	South Korea	0.02	170	10
8	Chung, Hyunsoo	South Korea	36	471	13	Lee, Jun Haeng	South Korea	0.03	168	22
9	Kim, Jie-Hyun	South Korea	35	391	19	Goto, Osamu	Japan	0.01	163	8
10	Lee, Jun Haeng	South Korea	34	1158	22	Abe, Seiichiro	Japan	0.02	162	16

### Analysis of references and cited references

**Figure 7:**
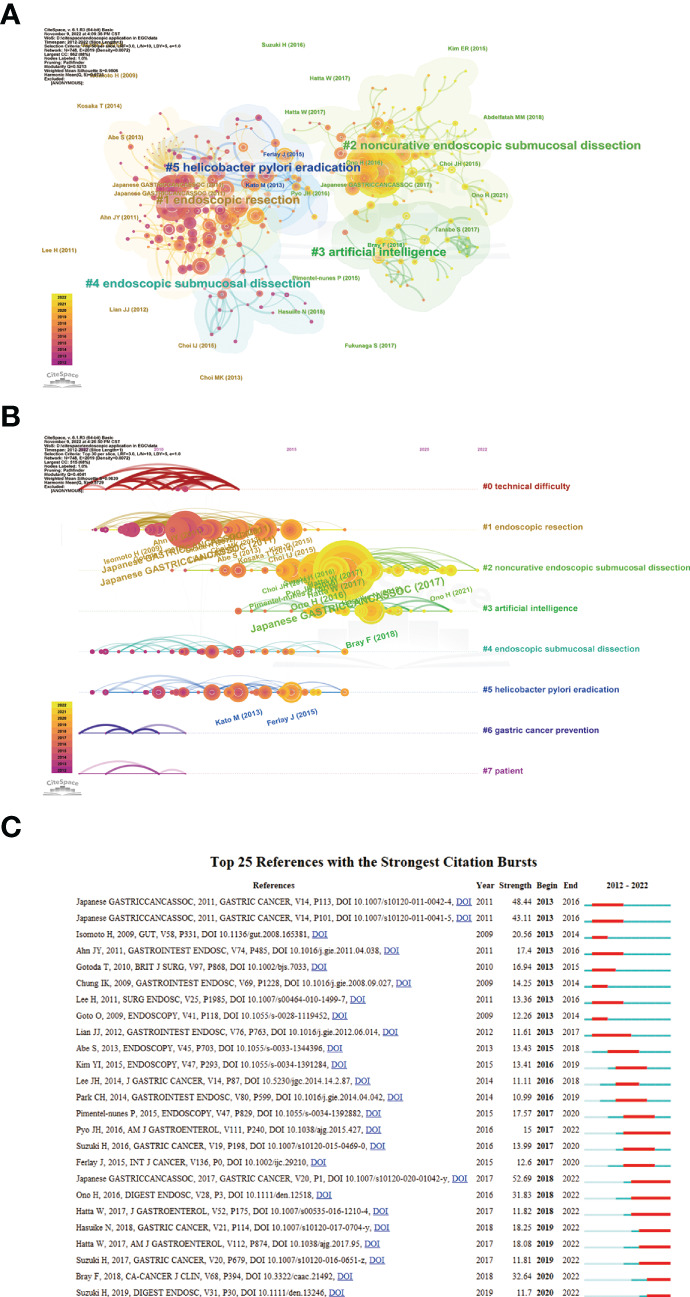
**(A)** A landscape view of co-cited reference cluster analysis. The area of diverse colors and shapes reflects the clusters, and node sizes denote cited times. **(B)** Visualization map of timeline view. The time evolution is reflected by various colored lines, and the nodes on the lines indicate the references cited. **(C)** CiteSpace visualization map of top 25 references with the strongest citation bursts from 2012 to 2022. Red bar indicates citation burst start and finish.

A total of 1,333 publications were included in this analysis, 78 of which had been cited at least 50 times. **Table 5** illustrates the top 10 articles with the largest number of citations. An article by Smyth E C et al. (4) had the largest number of citations (839), followed by those of Pimentel-Nunes P et al. (21) (737) and Ono H et al. (22) (343). Altogether, 17,453 cited references were included in our research (**Table 6**), with an article by Gotoda T et al. (23) being the most cited (416 citations), followed by two articles from the Japanese Gastric Cancer Association (371 and 261 citation, respectively) (24, 25). The co-citation network analysis of references is visually represented in **Figure 7A**. In terms of the analysis results, the modularity, *Q*, was 0.5213 (> 0.3), and the mean silhouette, *S*, was 0.9506 (> 0.7), revealing a considerable community structure and strong network homogeneity, respectively, and, therefore, the high reliability of the clustering results. The timeline view of co-citation references depicts the evolution of research hotspots over time (**Figure 7B**). Using this, we identified eight clusters that represent recent hotspots and research trends in endoscopic applications in the detection of EGC. The largest cluster was “non-curative endoscopic submucosal dissection” (cluster number 2), whereas “endoscopic resection” (cluster number 1) and “artificial intelligence” (cluster number 3) (AI) are the earliest and most recently identified hotspots in this field, respectively, suggesting that AI learning applied in EGC is gaining increasing interest. Half of the clusters, namely, “technical difficulty” (cluster number 0), “endoscopic resection” (cluster number 1), “non-curative endoscopic submucosal dissection” (cluster number 2), and “endoscopic submucosal dissection” (cluster number 4), are relevant to the endoscopic treatment applied in clinical settings. Citation bursts are phrases that appear abruptly or whose frequency of use increases substantially in a short period of time. In general, citation bursts illustrate the progression of a study field over time. **Figure 7C** depicts the top 25 references associated with the strongest citation bursts. The citation eruption for this particular research area began in 2013, and an article by Pyo J. H. et al. (26), about the controversial advantages and disadvantages of endoscopy and surgery in EGC treatment, was the most cited over a long period and is still cited frequently.

**Table 5:** Top 10 original articles related to endoscopic application in early gastric cancer.

Rank	Title	Journals	Author	Year	Citations
1	Gastric cancer	Lancet	Smyth, E. C; et al.	2020	839
2	Endoscopic submucosal dissection: European Society of Gastrointestinal Endoscopy (ESGE) Guideline	Endoscopy	Pimentel-Nunes, P; et al.	2015	737
3	Guidelines for endoscopic submucosal dissection and endoscopic mucosal resection for early gastric cancer	Digestive Endoscopy	Ono, H; et al.	2016	343
4	Helicobacter pylori Therapy for the Prevention of Metachronous Gastric Cancer	New England Journal of Medicine	Choi, I. J; et al.	2018	307
5	Diagnosis and management of iatrogenic endoscopic perforations: European Society of Gastrointestinal Endoscopy (ESGE) Position Statement	Endoscopic	Paspatis, G. A; et al.	2014	177
6	Scheduled endoscopic surveillance controls secondary cancer after curative endoscopic resection for early gastric cancer: a multicenter retrospective cohort study by Osaka University ESD study group	Gut	Kato, Motohiko; et al.	2013	161
7	Application of convolutional neural network in the diagnosis of the invasion depth of gastric cancer based on conventional endoscopy	Gastrointestinal Endoscopy	Zhu, Yan; et al.	2019	150
8	AGA Institute Clinical Practice Update: Endoscopic Submucosal Dissection in the United States	Clinical Gastroenterology and Hepatology	Draganov, P. V; et al.	2019	148
9	Complications of Gastric Endoscopic Submucosal Dissection	Digestive Endoscopy	Oda, I; et al.	2013	136
10	Endoscopic submucosal dissection	Gastrointestinal Endoscopy	Maple, J. T;et al	2015	135

**Table 6:** Top 10 co-cited references related to endoscopic application in early gastric cancer.

Rank	Cited reference	Journal	Citations	Authors	Year	DOI
1	Incidence of lymph node metastasis from early gastric cancer: estimation with a large number of cases at two large centers	Gastric Cancer	416	Gotoda, T; et al.	2000	10.1007/pl00011720
2	Japanese classification of gastric carcinoma: 3rd English edition	Gastric Cancer	371	Japanese Gastric Canc Assoc	2011	10.1007/s10120-011-0041-5
3	Japanese gastric cancer treatment guidelines 2014 (ver. 4)	Gastric Cancer	261	Japanese Gastric Canc Assoc	2017	10.1007/s10120-016-0622-4
4	Japanese gastric cancer treatment guidelines 2010 (ver. 3)	Gastric Cancer	257	Japanese Gastric Canc Assoc	2011	10.1007/s10120-011-0042-4
5	Endoscopic submucosal dissection for early gastric cancer: a large-scale feasibility study	Gut	209	Isomoto, H; et al.	2009	10.1136/gut.2008.165381
6	Endoscopic mucosal resection for treatment of early gastric cancer	Gut	208	Ono, H; et al.	2001	10.1136/gut.48.2.225
7	Incidence of lymph node metastasis and the feasibility of endoscopic resection for undifferentiated-type early gastric cancer	Gastric Cancer	172	Hirasawa, T; et al.	2009	10.1007/s10120-009-0515-x
8	Endoscopic resection of early gastric cancer	Gastric Cancer	166	Gotoda, T; et al.	2007	10.1007/s10120-006-0408-1
9	Guidelines for endoscopic submucosal dissection and endoscopic mucosal resection for early gastric cancer	Digestive Endoscopy	143	Ono, H; et al.	2016	10.1111/den.12518
10	Endoscopic submucosal dissection of early gastric cancer	Journal of Gastroenterology	142	Gotoda, T; et al.	2006	10.1007/s00535-006-1954-3

### Analysis of keywords

**Figure 8:**
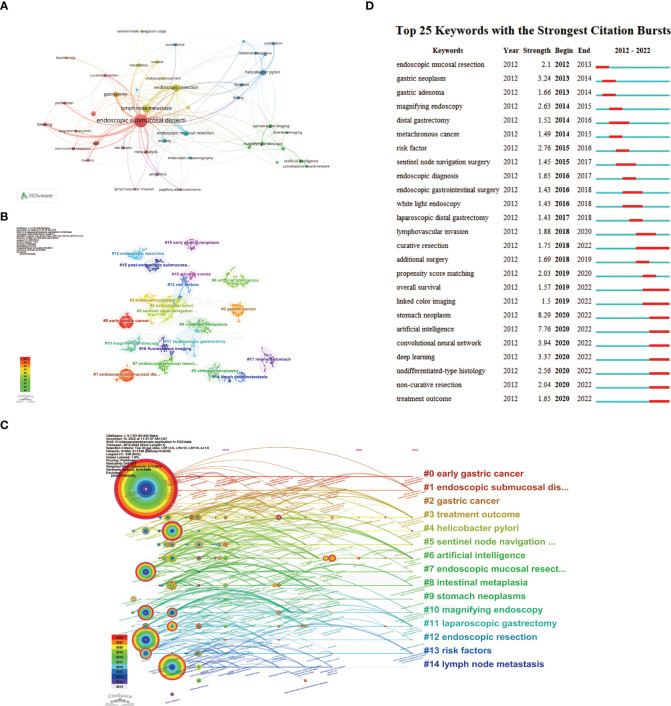
**(A)**. Network visualization map of the keywords occur more than 10 times, divided into 8 clusters with individual color. **(B)** A landscape view of keyword cluster analysis indicates the top 20 clusters. The different clusters are represented by different colors and shapes. **(C)** Timeline view visualization map of keywords, only images the top 15 clusters. The time evolution is indicated with different colored lines, and the nodes on the lines indicate the occurrence of the keyword. **(D)** Top 25 keywords with the strongest citation bursts from 2012 to 2022.

By using keyword co-occurrence analysis and citation burst detection, we may identify the most pertinent research hotspots and trends in recent years. Our study included a total of 1,652 author keywords, 51 of which had frequencies of over 10. The keywords’ network visualization map contains 43 keywords (**Figure 8A**), and shows that the phrase “endoscopic submucosal dissection” has the highest frequency and strong connections with other keywords such as “lymph node metastasis” and “bleeding”. **Table 7** shows the top 20 keywords with the highest frequencies. We used CiteSpace to create the keywords co-occurrence network (**Figure 8B**) and clusters were identified using a log-likelihood ratio (LLR) algorithm. As a result, 26 clusters emerged (**Supplementary Table 1**). The modularity value, *Q*, of 0.913 (> 0.3) and the mean silhouette value, *S*, of 0.9872 (> 0.7) of these clusters represent significant community structure and stable network homogeneity, demonstrating the high trustworthiness of the clustering results. These 26 clusters can be divided into six groups. Group 1 (cluster numbers 0, 2, 4, 8, 9, 16, 18, and 23) mainly revolves around the occurrence and development of EGC, including precancerous lesions, *Helicobacter pylori* infection and eradication, the application of proton pump inhibitors (PPIs), and early diagnosis. Group 2 (numbers 1, 3, 7, 12, 15, 18, 19, and 20) is concerned with ER treatment and its associated complications, including surgical methods and clinical outcomes of ESD and EMR, and adverse events such as post-ESD bleeding, pyloric stenosis, and gastrointestinal perforation. Group 3 (numbers 5, 11, 17, and 24) introduces minimally invasive surgery in EGC, basically involving sentinel node navigation surgery, laparoscopic and endoscopic cooperative surgery, non-curative endoscopic resection, and additional laparoscopic gastrectomy. Group 4 (numbers 13, 14, and 21) generalizes the risk factors and the nomograms used to predict lymph vascular invasion and lymph node metastasis. Group 5 (number 6) presents the various forms of AI applied in EGC diagnosis and treatment, such as convolutional neural networks (CNNs) and deep learning (DL). Group 6 (numbers 10, 16, and 25) recommends newly developed endoscopic technical applications in EGC, such as confocal laser endomicroscopy (CLE), endocytoscopy (EC), photodynamic diagnosis (PDD), and Raman spectroscopy.

**Table 7:** The top 20 keywords with the highest frequency related to endoscopic application in early gastric cancer.

Rank	Count	Centrality	Year	Keywords	Rank	Count	Centrality	Year	Keywords
1	458	0.53	2012	endoscopic submucosal dissection	11	20	0	2019	artificial intelligence
2	132	0.07	2013	lymph node metastasis	12	16	0.03	2014	signet ring cell carcinoma
3	107	0.13	2012	endoscopic resection	13	15	0.08	2013	intestinal metaplasia
4	62	0.43	2013	helicobacter pylori	14	14	0.25	2014	minimally invasive surgery
5	56	0.5	2012	endoscopic mucosal resection	15	13	0.13	2013	atrophic gastritis
6	39	0.32	2012	narrow-band imaging	16	13	0.08	2013	curative resection
7	24	0.44	2012	risk factor	17	13	0.16	2013	metachronous gastric cancer
8	23	0.09	2013	non-curative resection	18	12	0.09	2015	lymph node
9	22	0.4	2013	magnifying endoscopy	19	11	0	2019	convolutional neural network
10	21	0.1	2013	long-term outcome	20	11	0.09	2014	endoscopic treatment

The timeline view map illustrates the top 15 clusters, showing the progress made in this field from 2012 to 2022 (**Figure 8C**). The largest cluster was that of endoscopic submucosal dissection (number 1). The thicknesses of the red ring and outer purple ring indicate that this topic is still a research hotspot and occupies an unbeatable position in this field. The newest and most promising trend is AI (number 6). The keywords citation burst (**Figure 8D**) indicated that the earliest burst keyword was EMR and that curative resection had the longest burst time, but AI showed increasing burst intensity, suggesting that this topic will assume increasing importance in future research.

## Discussion

Research into endoscopy and EGC research has surged over the past decade; however, this is the first study utilizing BA (by means of CiteSpace and VOSviewer, two widely used literature mapping and visualization software packages) to characterize development patterns and hotspots in this field.

The steady increase in the average number of citations per year per document and volume of publications suggests that this discipline has reached its mature stage. The recent significant growth reflects interest in the new hotspots, which are largely related to the application of AI-assisted endoscopy in EGC. Japan, the Republic of Korea, and the People’s Republic of China place significant importance on this topic and have contributed to over half of the related publications. However, although the People’s Republic of China has achieved the third position in terms of the number of publications, its average number of citations per document was the lowest among the top 10 nations, indicating a dearth of high-quality papers that may be attributable to its late entry into this field. Research cooperation is usually based on geography and can be roughly divided into two groups: Asian and European countries. There is cooperation within the groups but insufficient cooperation between these two groups (**Figure 3D**).

Impact factor, JCR category, and other meaningful indicators were also utilized to evaluate journal quality. **Table 2** shows that *Gastric Cancer* has the largest total number of citations and *Endoscopy* has the largest average number of citations, illustrating their importance in this domain. *Gastrointestinal Endoscopy* and *Digestive Endoscopy* also have large average numbers of citations and high IFs. Based on this information, all of the abovementioned would be ideal journals for the publication of future studies. *Surgical Endoscopy and Other Interventional Techniques* has published the most articles, a fact that indicates that it has the potential, through the publication of higher-quality articles, to improve its academic reputation. The paths and circles in **Figure 4** illustrate the significance of cross-discipline collaboration to the advancement of this field. As illustrated in **Tables 2**–**4**, all of the top institutions, authors, and cited authors are from either Japan or the Republic of Korea, affirming these countries positions as field leaders. However, as can be seen in **Figure 5B** and **6B**, the connection between institutions and the connection between authors are weak. The People’s Republic of China, for example, is ranked third in terms of publication volume, but there is no Chinese institution or author in the top 10, indicating that, in this country, research pertinent to this field is still in its infancy. The People’s Republic of China could foster greater cooperation with other countries by enhancing the quality of its studies, which would enable it to make greater contributions to this field. Gotoda Takuji has the largest total number citations and highest degree of centrality among all co-cited authors, which is proof of the exceptional impact he has had in this field. His research focuses primarily on ER applied in the treatment of esophageal, gastric, and colonic malignancies, especially ESD of EGC (27).

The majority of the top 10 cited references (**Table 6**) are concentrated on the efficacy and safety of ER procedures used to treat EGC, EGC treatment guidelines, and the incidence of lymph node metastasis (LNM) in EGC. The co-cited reference clusters (**Figure 7B**) and the timeline map of keywords clusters (**Figure 8C**) indicate that the research hotspots have moved away from ER and *H. pylori* eradication to the application of AI in EGC. With this in mind, we combined these two analyses to further examine the trends in this field. The review that follows is concentrated on endoscopic diagnosis and treatment, based on the keywords and clusters identified by CiteSpace analysis (**Supplementary Table 1**).

### Endoscopic diagnosis

As a secondary strategy for gastric cancer prevention, endoscopy has played an important role in detecting preneoplastic conditions (28). Correa models indicate that, during the course of intestinal-type gastric carcinogenesis, the gastric mucosa changes from normal to non-atrophic gastritis to atrophic gastritis to intestinal metaplasia and, finally, to gastric cancer (29). However, from the “point of no return” (30) perspective, which entails identifying and treating curable lesions as early as possible through screening and surveillance endoscopy, *H. pylori* eradication may not reduce the risk of this gastric cancer cascade in patients with intestinal metaplasia and dysplasia. The optimal age and intervals for screening differ worldwide (31). Japan and the Republic of Korea have established national screening programs for years. In Japan, the guidelines stipulate a starting age of 50 years, after which endoscopic screening is offered biennially or triennially (32). In addition, a recent study (33) using microsimulation decision models to determine how the effectiveness and cost-effectiveness of national policies could be optimized recommends a triennial endoscopic screening for populations aged between 50 and 75 years. In the Republic of Korea, screening is performed on all individuals aged over 40 years at 2-year intervals (34), and Yun-Suhk Suh et al. (35) have demonstrated the cost-effectiveness of this program. Guidelines from the British Society of Gastroenterology recommend endoscopic screening in populations aged at least 50 years that have several risk factors for gastric adenocarcinoma (i.e., being male, a smoker, and having pernicious anemia), especially for individuals with a first-degree relative with gastric cancer, and endoscopic surveillance at 3-year intervals only for patients diagnosed with extensive atrophic gastritis or intestinal metaplasia (36).

White-light endoscopy (WLE) is a conventional and the most frequently used endoscopic technique in clinics, but it cannot always detect certain subtle morphological and color features (37). Because of its limited sensitivity, more advanced endoscopic imaging modalities that can be used for lesion detection have been developed (10). Chromoendoscopy necessitates the local application of stains or dyes to identify lesions from normal stomach mucosa. Narrow-band imaging (NBI) is a virtual and optical form of chromoendoscopy that can improve the visualization of the vascular network’s surface structure and contrast (8). Blue laser imaging (BLI) generates a far narrower blue spectrum without the filter required by NBI, resulting in increased light intensity, which is capable of identifying more entrenched surface and submucosal features (38). Magnifying endoscopy (ME) can better visualize the microstructures and microvessels of the lesions and is typically used in combination with another form of endoscopy (39). Image-enhanced endoscopy (IEE, such as NBI and BLI), paired with ME, has been shown to be effective in detecting EGC when compared with magnifying WLE (M-WLE) (40). A network meta-analysis of prospective studies illustrated that both ME-NBI and ME-BLI had a higher level of accuracy than either WLE [odds ratio (OR) 2.56 and 3.13, respectively] or M-WLI (OR 1.79 and 2.22, respectively); however, there was no significant difference between the EGC-diagnostic abilities of ME-NBI and ME-BLI (39).

Confocal laser endomicroscopy (CLE) is an innovative, cutting-edge technology that illuminates lesions with a low-power laser and collects reflected light in the same plane. Its light may be focused on the selected lesion depth, and its high (1,000-fold) magnification enables *in vivo* optical biopsy (41). The benefits of *in vivo* biopsy, such as its ability to identify margins of endoscopic resection and the optimal lesions for biopsy, to reduce the number of biopsies needed, and even to visualize *H. pylori*, to avoid biopsy altogether, are evident. These characteristics led to the establishment of the Miami classification of CLE criteria (42). The pooled sensitivity and specificity of CLE in the diagnosis of gastric cancer were 92% and 99%, respectively. CLE can also be used to discriminate differentiated and undifferentiated gastric cancer (43). However, if the procedure time of CLE is prolonged, sufficient sedation becomes essential. A retrospective cohort study (44) demonstrated that CLE with sedation was associated with significantly greater discrimination and lower misdiagnosis rates. The sensitivity and specificity of CLE for the diagnosis of EGC in sedated subjects were 1.00 and 0.95, respectively, compared with 0.89 and 0.88, respectively, in non-sedated subjects (44). A recent meta-analysis found that the pooled sensitivity and specificity of CLE in detecting focal precancerous lesions of gastric cancer were 90% and 87%, respectively, slightly higher than for NBI (87% and 85%) (45). Al−Mansour et al. (41) observed that CLE can improve the accuracy of EGC diagnosis, as well as reduce the incidence of rare adverse effects primarily associated with the use of contrast dyes. Nevertheless, it has distinct limitations: considerable time is required for image acquisition, there is a steep learning curve associated with image interpretation, and the required equipment is typically available only at tertiary care centers (46).

Endocytoscopy (EC) is one of the most sophisticated current techniques, based on ultra-high magnification endoscopy, and allows the *in vivo* microscopic visualization of the surface level of mucosal cells with the use of stains (8, 47). Fourth-generation endocytoscopes support 500× continuous zoom-focus magnification, and the average time needed to conduct EC is approximately 10–20 minutes (48). Standardized classification criteria for EC-detected stomach cancer are not yet in place; however, in another study, Inoue Haruhiro et al. (8, 48, 49) developed a simple, three-level, classification of EC-detected colorectal lesions (50): non-neoplastic lesions are classified as EC1, adenoma is classified as EC2, and carcinoma is classified as EC3 (8). Furthermore, they identified an “enlarged nuclear sign”(ENS) as a distinct characteristic of gastric adenocarcinomas and reported that the sensitivity, specificity, and accuracy of ENS for the diagnosis of EGC were 84.8%, 90.0%, and 87.2%, respectively (49). One meta-analysis that included all research on the use of endocytoscopy in the diagnosis of EGC, conducted before November 2021, found that the pooled sensitivity, specificity, and accuracy were 83.52%, 91.7%, and 89.2%, respectively (51). However, some factors limit the widespread application of EC. First, abundant gastric secretions can lead to the EC images being poor in quality; however, Abad MRA et al. (49) reported that the likelihood of obtaining high-quality images can be increased by up to 80% by using the anti-foaming agent dimethicone and a water-based mixture containing the mucolytic pronase before the operation, and by repeated careful water-jet-assisted mucosal rinsing prior to the double staining of CM (49). Second, unlike fluorescence-based CLE, EC cannot detect the depth of the gastric lesion invasion because it can detect only the superficial epithelial layer. In addition, EC is expensive and requires the operator to be fully trained, with the result that it is carried out in only a few facilities throughout the world.

There are two types of endoscopic ultrasonography (EUS): conventional EUS and miniprobe EUS. Miniprobe EUS has better accuracy in the detection of small and superficial tumors (52). The major applications of EUS in EGC involve predicting the invasion depth to distinguish the T stages and the detection of nodal disease before endoscopic resection (ER) (10); however, the efficacy of these two applications remains debatable. In previously published guidelines, EUS was the technique recommended for the determination of the extent of the tumor and assessment of the T stage (53), and as a complementary diagnostic technique to conventional endoscopy (51). Jung Kim et al. (52) retrospectively analyzed 6,084 patients and reported that the accuracy of EUS and EGC in the detection of T1a was 75.0% and 89.4%, respectively, and that the accuracy of miniprobe EUS in its detection of T1a with lesions ≤ 2 cm in size, and of conventional EUS in its detection of T1a with lesions > 2 cm in size, was 84.6% and 83.2%, respectively. An *in vitro* study reported that EUS is suitable for pretreatment T−staging and standardized operation procedures and that high-quality images can increase its accuracy (54). However, the European Society of Gastrointestinal Endoscopy’s (ESGE) recently revised guidelines do not recommend routinely applying EUS before ER, because of its high rates of erroneous staging and misdiagnosis (55). A meta-statis with 3,401 patients also reported that the diagnostic ability of EUS was limited, that there was significant heterogeneity among studies, such as the diagnostic accuracy in China is significantly lower than that in Japan and Korea population, indicated that ethnicity could influence diagnostic accuracy (56). For the diagnosis of nodal disease, which is typically carried out before ER, EUS was also associated with variable accuracy (10). Hua Zhou et al. (57) reported that the depth of lesion invasion may affect the diagnostic accuracy of EUS, and that, in general, its accuracy needed to be improved. We expect that future research will determine the effectiveness of EUS in EGC applications.

In addition to the aforementioned different types of endoscopies, other approaches can be integrated into screening and surveillance endoscopy. As an optical diagnostic method for identifying tumors, photodynamic diagnosis (PDD) involves first the administration of specific photosensitizers (PSs), including verteporfin, talaporfin, and 5-aminolevulinic acid (5-ALA), among which 5-ALA is the most suitable and widely used (58). These subsequently accumulate in tumors, and can be illuminated with light of a specific wavelength, enabling visualization of the tumors by fluorescence (59). Raman spectroscopy is a molecular vibrational spectroscopy method that may also be used to diagnose malignancies *in vivo*. The characteristic peaks and intensity of Raman spectra reflect the quantity and composition of chemical substances produced by gastric lesions during the process of carcinogenesis (60). This technique has a pooled sensitivity and specificity of 0.89 and 0.92, respectively (61). Therefore, the use of endoscopy paired with fiber-optic Raman spectroscopy may allow the simultaneous observation of tumors and generation of Raman spectral data, in turn enabling real-time diagnosis (62). These two procedures have the potential to be objective diagnostic tools for the diagnosis of EGC, but more prospective studies to prove their usefulness are still needed.

The keyword “*Helicobacter pylori*” was also important in our analysis. It has been shown that *H. pylori* eradication reduces the incidence of GC by only 33%–47% (63), and a meta-analysis reported that *H. pylori* eradication did not benefit patients with intestinal metaplasia or dysplasia, who were at risk of developing GC (64), which indicates the importance of undertaking endoscopic surveillance for gastric cancer after eradication therapy (GCAE). Based on prior research, a recent global consensus (65) states that surveillance at an interval of 2–3 years should be carried out in patients with advanced gastric atrophy or intestinal metaplasia after *H. pylori* eradication. In terms of GCAE’s histological features, Masanori Ito et al. (66) reported that GCAE lesions were covered with normal or mildly atypical epithelium, which they named epithelium with low-grade atypia (ELA), and other researchers have termed “nonneoplastic epithelium” (67), and that can prevent the early endoscopic diagnosis of GCAE. The endoscopic characteristics of GCAE include a gastritis-like appearance and reddish depressions. However, limited research suggests that ME-NBI, BLE, and linked-color imaging may be better suited to the detection of GCAE than WLE (68–70).

“Artificial intelligence” is the newest hotspot among all clusters, and includes several keywords, such as “deep learning (DL)” and “convolutional neural network (CNN)”. DL as a subcategory of machine learning is inspired by the human brain’s biological neural network, which can rationally process data, draw conclusions, and make decisions (71). After training, DL algorithms show considerable capacity for image recognition (72). CNN is a specific DL method, the structure of which mimics the organization of the brain’s visual cortex and is therefore particularly suited to image recognition and video analysis tasks. Cancer detection and prediction are the primary AI topics in EGC research. Computer-assisted detection (CADe) and computer-assisted diagnosis (CADx) are the two major AI systems used in the diagnosis of EGC. CADe is usually used for the detection and location of suspected lesions and whose outputs are lesion outlines in the form of circles or squares; CADx is generally used for diagnosis, as it can predict the depth of lesion invasions and differentiate between neoplastic and non-neoplastic tissues, and identify precancerous conditions. Combining these two systems can help endoscopists to target tissue suitable for biopsy (73). Toshiaki Hirasawa et al. (74) first reported applying CNN to detect GC in still images, with an overall sensitivity of 92.2% and a positive predictive value of 30.6%. Subsequently, numerous relevant studies have been published, and a meta-analysis of 15 studies published prior to November 2021 reported a pooled sensitivity and specificity of 0.87 and 0.88, respectively; a subgroup analysis found that the sensitivity and specificity of AI-assisted WLE were 0.86 and 0.87, respectively, whereas the sensitivity and specificity of AI-assisted NBI were 0.97 and 0.96, respectively (75). Ling T et al. (76) reported that the sensitivity and specificity of their AI system in delineating tumor margins in real-time video was 82.7% in the case of differentiated GC and 88.1% in the case of undifferentiated GC; both scores were higher than those achieved by human experts. Recently, the accuracy of Ling T et al.’s AI system in clinical trials has also been examined. A series of articles on the system created by Wuhan University, “ENDOANGEL”, has been published (73), and the results of the latest multicenter clinical trial show that the sensitivity and accuracy of the system are 92.59% and 83.67%, respectively (77). A competition was held to compare the performance of “ENDOANGEL” with that of endoscopists, and the results showed that the sensitivity of “ENDOANGEL” for the diagnosis of EGC, prediction of EGC invasion depth, and determination of differentiation status was 100%, 78.57%, and 71.43%, respectively. These values are all significantly higher than those achieved by endoscopists (87.13%, 63.75%, and 64.41%, respectively) (78). Another AI model, created by Nam J Y et al. (79), was shown to have a similar performance. Atsushi Goto et al. (80) designed an AI classifier to assist endoscopists in differentiating between intramucosal and submucosal GC. The accuracy, sensitivity, and specificity of this cooperative technique were 78%, 76%, and 80%, respectively, substantially better than those of the AI- or endoscopists-only models.In addition to their use in tumor detection, the application of AI models to predict patients’ pre- and post-operative condition has also advanced in recent years. Hae-Ryong Yun et al. (81) created an ML model (XGBoost model) to predict non-curative resection of EGC before ESD, which had sensitivity, specificity, and area under the receiver operating characteristic (AUROC) values of 0.785, 0.732, and 0.851, respectively. In general, AI provides significant benefits; for example, it is not affected by human error, fatigue, or inattention, and it has high diagnostic accuracy. However, more research is needed to ensure its accuracy in clinical settings and establish a fully functional predict–diagnosis–treat system. A transition period, in which endoscopists can become familiar with this newly developed technique, is also required.

### Endoscopic treatment

Endoscopy resection (ER) has become an integral part of EGC treatment, accounting for nearly 60% of EGC therapy in Japan. Based on the recently published guidelines in Japan, ESD has more indications than EMR, ESD can be applied to any size of intramucosal (cT1a) differentiated-type EGC, to cT1a differentiated-type EGC ≤ 3 cm in diameter, and to cT1a undifferentiated-type EGC ≤ 2 cm in diameter, without causing ulceration or ulcer scarring. By contrast, it is advised that EMR is applied only to cT1a differentiated-type EGC ≤ 2 cm in diameter when again it causes no ulceration or ulcer scarring (9). According to the latest ESGE guidelines, EMR is an alternative technique and should be used only on lesions that are elevated by less than 1 cm and have a low likelihood of progression (55). It is worth noting that this differs from information given in the first edition of the guidelines, published in 2016 (22), which stated, as mentioned above, that “expanded indications for ESD/EMR have been integrated into the “absolute indications”. The expanded indications referred to here are cT1a differentiated- type cancers locally recurring after endoscopic resection of lesions for which ESD/EMR is absolutely indicated (9). Compared with EMR, ESD is associated with higher rates of complete resection, curative resection, en bloc resection, and local recurrence, although operation times are longer and rates of perforation are higher (but not so high as to be a cause for concern) (82). These findings make clear that ESD should be the primary choice for the treatment of superficial gastric lesions (55). No research has conclusively demonstrated the difference between ESD and surgery in terms of prognosis or quality of life (9). ESD is also associated with shorter operation times and hospital stays, and a lower likelihood of adverse events and procedure-related mortality, but with higher recurrence rates, lower disease-free survival rates, and a larger number of metachronous lesions (55). After ER, endoscopic surveillance carried out at 1-year intervals is recommended to detect local recurrence and metachronous lesions. Patients with multiple lesions, precancerous conditions, and *H. pylori* infections, and elderly patients, have a higher risk of developing metachronous lesions (9, 55).

In contrast to surgeons, endoscopists cannot provide direct hand-assisted traction to lesions, which means that endoscopists may benefit from proper traction methods for improved direction, submucosal visibility, and force application (83). Multiple methods to achieve this have been designed in this century. Clip line traction is the most common traction method, but it has been demonstrated that dental floss clip (DFC) line traction is associated with shorter procedure times, especially for tissue located in the greater curvature of the upper or middle stomach. Water-pocket creation ESD (WP-ESD) has also been shown to have a significantly shorter procedure time than conventional ESD, particularly for tissue located in the middle and lower thirds of the stomach (84). Other methods, including double scope, sheath, elastic band, and spring-and-loop clip, have also been proven to have shorter procedure times than conventional ESD. However, as the stomach has a thicker mucosal layer and offers a larger working space for endoscopies than the esophagus or colon, Seiichiro Abe et al. (84) stated that the above mentioned traction methods should serve as alternative choices to conventional ESD, instead of the routine choice.

During and after ER, complications, particularly bleeding and perforation, can occur. A multicenter prospective study with 9,616 patients found rates of postoperative bleeding, intraoperative perforation, and delayed perforation of 4.4%, 2.3%, and 0.4%, respectively. Among those patients with complications, the rates of blood transfusion and emergency surgery were 10.1% and 3.4%, respectively (85). The risk of postoperative bleeding was comparable between ESD and EMR (10). Guidelines in Japan (9) recommend the implementation of preventive measures, such as coagulating visible remnant vessels and administering gastric acid secretion inhibitors, to reduce postoperative bleeding rates [in this context, PPIs were found to be superior to H_2_-receptor antagonists (H_2_RAs)]. In addition, recent nationwide prospective studies have demonstrated that vonoprazan, a novel potassium-competitive acid blocker (P-CAB), is associated with a lower risk of delayed bleeding than PPIs and reported that it may reduce the risk of post-ESD bleeding by 30%, as well as reducing rates of blood transfusion and perforation (86, 87). By contrast, routine second-look endoscopy was not found to reduce bleeding rates and was recently shown to be ineffective for patients administered antithrombotic agents (88). Perforation is more likely to occur during ESD than EMR (82), and during the operations, the primary strategy used to reduce the risk of this is endoscopic closure (9). In addition to macroscopic perforations, micro-perforation or suspected perforation can be managed with fasting, intravenous antibiotics, or additional endoscopic clip closure (10). Post-ESD stenosis usually occurs after resecting gastric antral or cardia lesions, and resections of a circumference over three-quarters the length of tissue are a significant risk factor for this. Endoscopic balloon dilatation (EBD) is the primary strategy for stenosis management. Some articles have indicated that steroids, administered orally or by injection, may be effective in preventing stenosis, but the use of these remains controversial (89).

Lymph node metastasis is key to assessing the disease progression and prognosis of EGC patients (90). Although the presence of LNM is not necessary for the diagnosis of EGC, it is a prerequisite for ER. If the presence of LNM is confirmed, either before or after ER, either ER will not be the primary operation method used or additional surgery will be required. It is difficult to preoperatively diagnose LNM with currently utilized methods such as WLE and EUS. Therefore, recent studies propose using nomograms, which are constructed based on multivariable factors such as age, tumor size, depth of invasion, histological subtype, and even human epidermal growth factor receptor 2 (HER2), to identify LNM. These studies have shown that nomograms have great accuracy and clinical performance (91–93). After non-curative resection, it is vital to confirm that LNM is absent, as this will determine whether or not additional surgery for post-ER patients is required. A recently published meta-analysis indicated that the risk of LNM was significantly increased in tumors that were > 30 mm in size, had an invasion depth of ≥ 500 μm, flat or elevated tumor macroscopic appearance, and were of the undifferentiated histopathological type, after resection, had positive vertical margins and indications of lymphovascular invasion (LVI) (94). Another meta-analysis reached similar conclusions and specifically pointed out that lymphatic invasion and vascular invasion individually, rather than LVI, should be assessed, because vascular invasion did not show a significant statistical difference (95).

The application of endoscopy in EGC has improved significantly during the past decade. Japan and the Republic of Korea have established national strategies for EGC screening and surveillance, and their impressive results should encourage other countries/regions to establish their own EGC prevention programs. As regards endoscopic diagnosis, several types of endoscopy and auxiliary methods, such as endocytoscopy and photodynamic diagnosis, have been evaluated and implemented in clinical settings. Future researchers should concentrate on overcoming the limitations associated with the increased use of these techniques and on performing further clinical trials. Endoscopic therapy focuses mostly on ER indications, traction methods, post-ER complications, and LNM assessment. Recently published guidelines included a larger number of absolute indications than was previously identified; we believe that future research will broaden these indications based on newly discovered techniques and more precise clinical trials. Over the last decade, the research emphasis of this field has shifted from ER to AI. Recently developed AI systems have proved their distinct benefits in EGC diagnosis and forecasting. The “robot-assisted ESD” technique has been evaluated in a small number of studies and has tremendous promise; however, more clinical trials are required to justify the use of AI in EGC treatment. Regardless of its associated limitations and difficulties, the future of AI as it is applied in EGC is bright.

### Limitations

Our research has several limitations. First, the length of this text prevents us from expanding on other pertinent issues, such as intraoperative endoscopy for EGC localization to facilitate laparoscopic gastrectomy. Second, we retrieved only literature published in English from the Web of Science (WoS), and it would be preferable to integrate other language studies and databases. Last, but not least, it takes time for recently published high-quality publications to gain sufficient citations and establish hotspots, and some such articles may have been overlooked in our research.

## Conclusion

In conclusion, endoscopy is the most important technique for the diagnosis and treatment of EGC. The volume of relevant research has increased steadily in the last decade, with Japan and the Republic of Korea being the core countries that account for the most publications, high-yield institutions, and authors. Endoscopic submucosal dissection and AI are the major and most recently identified research hotspots in this field. This study provides a global overview of endoscopy applied in EGC, and briefly summarizes the recent developments in this field, identified by on the keywords we detected by BA.

## Author contributions

YL and SD conceived and designed the study. YL and HW conducted the statistical analyses. YL, HW, and QW drafted the manuscript. YL and SD consulted and supplemented the relevant information. YL and WH had full access to all the data and took responsibility for the integrity of the data and the accuracy of the data analysis. QW reviewed and edited the manuscript for content and style and polished the English content of this publication. All authors participated in the interpretation of the results. All authors contributed to the article and approved the submitted version.
