# Supplementary material for: Research trends in endoscopic applications in early gastric cancer: A bibliometric analysis of studies published from 2012 to 2022
Source: Front Oncol. 2023 Apr 11;13:1124498. doi: 10.3389/fonc.2023.1124498 (PMC10129370; doi:10.3389/fonc.2023.1124498)
Supplement: Supplementary file 1 [file Table_1.docx]

**SUPPLEMENTARY TABLE 1 |** Keywords clusters

| **ClusterID** | **Cluster** | **Size** | **Silhouette** | **Mean (Year)** | **Label (LSI)** | **Label (LLR)** | **Label (MI)** |
| --- | --- | --- | --- | --- | --- | --- | --- |
| 0 | early gastric cancer | 83 | 1 | 2016 | gastric cancer;  gastric epithelial neoplasm;  adjuvant chemotherapy;  elderly patients;  endoscopic gastric atrophy classification;  endoscopic submucosal dissection;  adjuvant chemotherapy;  elderly patients;  endoscopic gastric atrophy classification;  expanded indication | early gastric cancer (71.47, 1.0E-4);  gastric cancer (49.54, 1.0E-4);  helicobacter pylori (9.74, 0.005);  early gastric cancer (egc) (6.53, 0.05);  stomach neoplasms (6.38, 0.05) | adjuvant chemotherapy (1.14);  accuracy (1.14); ves |
| 1 | endoscopic submucosal dissection; | 80 | 0.986 | 2017 | endoscopic submucosal dissection;  early-stage gastric cancer;  intraepithelial neoplasia;  high grade;  preoperative evaluation;  gastric cancer;  endoscopic features;  charlson comorbidity index;  prognostic nutritional index;  elderly patients | endoscopic submucosal dissection (83.52, 1.0E-4);  gastric cancer (14.6, 0.001);  propofol (10.19, 0.005);  endoscopic resection (9.6, 0.005);  traction method (6.79, 0.01) | natural orifice endoscopic surgery/instrumentation |
| 2 | gastric cancer | 59 | 1 | 2016 | gastric cancer;  endoscopic submucosal dissection; hepatocellular carcinoma;  chronic hepatitis b;  liver cirrhosis;  cancer stem cell;  lymph node metastasis;  epithelial mesenchymal transition; laparoscopic sentinel lymph node dissection;  endoscopic full-thickness gastric resection | gastric cancer (140.47, 1.0E-4);  early gastric cancer (44.24, 1.0E-4); chemotherapy (10.17, 0.005); endoscopic procedure (9.64, 0.005); cancer stem cell (9.64, 0.005) | natural orifice endoscopic surgery/instrumentation |
| 3 | treatment outcome | 52 | 0.99 | 2016 | endoscopic submucosal dissection; curative resection;  lymph node metastasis;  ecura system;  esophagogastric junction;  gastric cancer;  gastrointestinal endoscopy;  mixed carcinoma;  retrospective studies;  antiplatelet agents | treatment outcome (21.38, 1.0E-4);  case report (20.42, 1.0E-4); gastrointestinal endoscopy (18.97, 1.0E-4);  systematic review (18.97, 1.0E-4);  curative resection (16.03, 1.0E-4) | quality indicators (0.16); gene mutation (0.16); u |
| 4 | helicobacter pylori; | 51 | 0.976 | 2015 | helicobacter pylori;  post-marketing surveillance study;  eradication rate;  gastric cancer-related death;  liver metastasis;  gastric cancer;  raman spectroscopy;  helicobacter pylori;  comparative study;  sclerosing cholecystocholangitis | helicobacter pylori (46.45, 1.0E-4); proton pump inhibitor (20.17, 1.0E-4); gastric carcinogenesis (13.42, 0.001); elimination of gastric cancer (13.42, 0.001); ulcer healing (9.68, 0.005) | rebamipide -artificial ulcer (0.13); gastroscopic |
| 5 | sentinel node navigation surgery | 45 | 0.976 | 2015 | gastric cancer;  laparoscopic treatment;  sentinel lymph node navigation;  sentinel lymph node basin;  submucosal tunnel;  invasive surgery;  sentinel node navigation surgery;  local resection;  esophageal cancer;  endoscopic submucosal dissection | sentinel node navigation surgery  (35.44, 1.0E-4);  minimally invasive surgery (30.1, 1.0E-4);  colorectal cancer (16.75, 1.0E-4);  esophageal cancer (14.58, 0.001);  function-preserving gastrectomy (14.1, 0.001) | surgical diagnostic technique (0.1); selective lym |
| 6 | artificial intelligence | 40 | 0.976 | 2016 | gastric cancer;  endoscopic ultrasonography;  submucosal deformity;  convolutional neural network;  hybrid laparoscopic;  artificial intelligence;  convolutional neural network;  submucosal deformity;  hybrid laparoscopic;  real-world evidence | artificial intelligence (40.66, 1.0E-4); convolutional neural network (39.9, 1.0E-4); deep learning (27.87, 1.0E-4);  submucosal tumor (26.52, 1.0E-4); endoscopic ultrasound (19.86, 1.0E-4) | superficial cancer (0.14);  real-world evidence (0. |
| 7 | endoscopic mucosal resection | 39 | 0.998 | 2015 | endoscopic mucosal resection; endoscopic submucosal dissection; gastric cancer;  pathological staging;  esophageal cancer;  colorectal cancer;  resection criteria;  submucosal dissection;  esophageal cancer;  endoscopic ablation | endoscopic mucosal resection  (49.38, 1.0E-4);  early colorectal cancer (13.68, 0.001);  early esophageal cancer (13.68, 0.001); endoscopic mucosal resection (emr)  (8.27, 0.005);  complications (7.15, 0.01) | topography lymph nodes (0.12);  neuroendocrine tumo |
| 8 | intestinal metaplasia | 39 | 0.984 | 2015 | gastric cancer;  gastric atrophy;  narrow-band imaging;  autofluorescence imaging;  helicobacter pylori;  intestinal metaplasia;  atrophic gastritis;  endoscopic submucosal dissection;  gastric dysplasia;  helicobacter pylori | intestinal metaplasia (36.63, 1.0E-4);  atrophic gastritis (36.63, 1.0E-4);  gastric atrophy (23.17, 1.0E-4);  blue laser imaging (21.04, 1.0E-4);  linked color imaging (21.04, 1.0E-4) | visibility (0.11);  mirna (0.11);  vessel density (0 |
| 9 | stomach neoplasms | 39 | 0.986 | 2015 | stomach neoplasms;  neoplasm staging;  patient selection;  positron-emission tomography;  screening program;  endoscopic submucosal dissection; stomach neoplasm;  ecabet sodium;  stomach ulcer;  helicobacter pylori | stomach neoplasms (82.49, 1.0E-4);  stomach neoplasm (19.95, 1.0E-4);  early diagnosis (18.62, 1.0E-4);  follow-up studies (18.62, 1.0E-4);  early gastric cancer (16.98, 1.0E-4) | angiodysplasia (0.18); utility (0.18);  outcome ass |
| 10 | magnifying endoscopy | 35 | 0.99 | 2016 | gastric cancer;  narrow-band imaging;  subepithelial capillary;  horizontal margin;  intervening part;  narrow band imaging;  acetic acid;  helicobacter pylori;  high definition endoscopy;  intervening part | magnifying endoscopy (37.63, 1.0E-4);  narrow-band imaging (24.63, 1.0E-4);  narrow band imaging (23.07, 1.0E-4); endocytoscopy (20.97, 1.0E-4);  confocal laser endomicroscopy  (16.54, 1.0E-4) | human h-ferritin (0.11); white light imaging endos |
| 11 | laparoscopic gastrectomy | 33 | 0.987 | 2016 | gastric cancer;  endoscopic submucosal dissection;  non-curative resection;  indication criteria;  long-term survival;  elderly patients;  prognostic factors;  charlson comorbidity index;  non-anesthesiologist administrated propofol;  asa classification | laparoscopic gastrectomy (22.25, 1.0E-4); elderly patients (16.55, 1.0E-4);  additional surgery (15.62, 1.0E-4);  non-curative endoscopic resection  (14.8, 0.001);  non-curative resection (11.91, 0.001) | in-hospital mortality (0.08);  antithrombotic drug |
| 12 | endoscopic resection | 32 | 0.999 | 2015 | endoscopic resection;  gastrointestinal disease;  precancerous conditions;  cancerous lesions;  lymph node metastasis;  gastric cancer;  endoscopic submucosal dissection; gastric tube cancer;  artificial ulcer closure;  prophylactic endoscopic balloon dilation | endoscopic resection (68.8, 1.0E-4);  gastric stenosis (12.34, 0.001);  endoscopic balloon dilation (ebd)  (12.34, 0.001);  extragastric recurrence (12.34, 0.001);  sfrp4 (6.16, 0.05) | sfrp4 (0.18);  single patient classifier genes (0.1 |
| 13 | risk factors | 30 | 0.982 | 2016 | gastric cancer;  endoscopic submucosal dissection; lymphovascular invasion;  positive vertical margins;  incomplete resection;  lymph node metastasis;  lymphatic involvement;  data-mining analysis;  undifferentiated-type early gastric cancer; residual tumors | risk factors (20.08, 1.0E-4);  lymphovascular invasion (15.45, 1.0E-4); endoscopic therapy (14.69, 0.001);  residual tumor (14.69, 0.001);  lymphatic involvement (10.92, 0.001) | positive vertical margins (0.09);  additional posto |
| 14 | lymph node metastasis | 27 | 0.988 | 2015 | lymph node metastasis;  gastric cancer;  endoscopic submucosal dissection; submucosal invasion;  submucosal invasion depth;  gastric carcinoma;  gastric net;  microsatellite instability;  neoplasm staging;  risk stratification | lymph node metastasis (70.76, 1.0E-4); endoscopic treatment (23.19, 1.0E-4); histological type (12.67, 0.001);  nomogram (10.56, 0.005);  surgical treatment (7.66, 0.01) | tumor markers (0.24); poorly differentiated cancer |
| 15 | post-endoscopic submucosal dissection bleeding | 26 | 0.995 | 2017 | post-endoscopic submucosal dissection bleeding;  coating area;  device delivery station system; polyglycolic acid sheet;  submucosal invasion;  endoscopic submucosal dissection;  food residue;  post-esd bleeding;  deep learning model;  ulcerative early gastric cancer | post-endoscopic submucosal dissection bleeding (14.02, 0.001);  gastric emptying (12.32, 0.001);  submucosal invasion (12.32, 0.001);  delivery of polyglycolic acid sheet  (8.88, 0.005);  lesser curvature (8.88, 0.005) | early gastric cancer (0.08);  endoscopic submucosal |
| 16 | fluorescence imaging | 21 | 0.937 | 2015 | gastric cancer;  endoscopic submucosal dissection; gastric adenoma;  reddish change;  acetic acid indigocarmine mixture;  fluorescence imaging;  photodynamic therapy;  photodynamic diagnosis;  5-aminolevulinic acid;  reddish change | fluorescence imaging (19.5, 1.0E-4);  gastric adenoma (19.5, 1.0E-4);  snare polypectomy (9.7, 0.005); photodynamic diagnosis (9.7, 0.005); duodenal adenoma (9.7, 0.005) | early gastric cancer (0.08);  endoscopic submucosal |
| 17 | remnant stomach | 18 | 0.978 | 2016 | gastric cancer;  distal gastrectomy;  tumor localization;  gastric adenocarcinoma;  intraoperative gastroscopy;  remnant stomach;  anastomosis site;  gastric cancer; remnant gastric cancer; endoscopic submucosal dissection | remnant stomach (23.38, 1.0E-4);  distal gastrectomy (23.38, 1.0E-4); anastomosis site (14.7, 0.001);  gastric tube (9.21, 0.005);  intraoperative gastroscopy (9.21, 0.005) | early gastric cancer (0.08);  endoscopic submucosal |
| 18 | early gastric neoplasm | 15 | 0.995 | 2017 | endoscopic submucosal dissection; gastric neoplasm;  second-look endoscopy;  recurrent bleeding;  forrest classification;  lansoprazole od;  delayed bleeding;  gastric tumor;  non-inferiority test;  prediction model | early gastric neoplasm (19.91, 1.0E-4); recurrent bleeding (9.9, 0.005);  fibrin glue (9.9, 0.005);  endoscopic for-ceps biopsy (9.9, 0.005);  non-inferiority test (9.9, 0.005) | early gastric cancer (0.09);  endoscopic submucosal |
| 19 | adverse events | 13 | 1 | 2014 | adverse events;  pyloric stenosis;  local triamcinolone acetonide injection; cytomegalovirus-associated ulcer;  local immunosuppression;  endoscopic submucosal dissection; expanded criteria;  local recurrence;  distant metastasis; gastric cancer | adverse events (21.13, 1.0E-4);  gastric esd (10.49, 0.005);  hemostatic time (10.49, 0.005);  pyloric stenosis (10.49, 0.005);  local triamcinolone acetonide injection (10.49, 0.005) | early gastric cancer (0.09);  endoscopic submucosal |
| 20 | endoscopic submucosal dissection complications | 13 | 0.997 | 2013 | endoscopic submucosal dissection; endoscopic submucosal dissection training;  endoscopic submucosal dissection techniques;  gastrointestinal neoplasias;  learning curve;  gastrointestinal perforation; gastrointestinal fistulae;  the-scope clip;  gastrointestinal bleeding;  clinical tutoring | endoscopic submucosal dissection complications (17.94, 1.0E-4); gastrointestinal perforation (10.79, 0.005); gastrointestinal fistulae (10.79, 0.005); gastrointestinal bleeding (10.79, 0.005);  over-the-scope clip (10.79, 0.005) | early gastric cancer (0.09);  endoscopic submucosal |
| 21 | signet ring cell carcinomal | 12 | 0.943 | 2017 | gastric cancer;  signet ring cell carcinoma;  cohesive carcinoma;  helicobacter pylori;  microsatellite instability;  endoscopic gastrointestinal surgery; helicobacter pylori;  undifferentiated-type carcinoma;  mixed type;  endoscopic resection | signet ring cell carcinoma (14.64, 0.001); lymph node (13.45, 0.001);  endoscopic gastrointestinal surgery  (13.45, 0.001);  lymph nodes (10.59, 0.005);  metastasis (10.59, 0.005) | early gastric cancer (0.07);  endoscopic submucosal |
| 22 | differentiated type | 11 | 0.991 | 2015 | gastric cancer;  mixed histology;  endoscopic submucosal dissection; undifferentiated adenocarcinoma; additional indication;  mucosal gastric cancer;  submucosal gastric cancer;  gastric carcinoma;  endoscopic resection;  mixed histology | differentiated type (10.63, 0.005); undifferentiated adenocarcinoma  (10.63, 0.005);  mixed type (7.87, 0.01);  submucosal gastric cancer (7.87, 0.01); mucosal gastric cancer (7.87, 0.01) | early gastric cancer (0.09);  endoscopic submucosal |
| 23 | h. pylori | 10 | 1 | 2016 | narrow band imaging;  gastric cancer;  diagnostic reliability;  white light endoscopy;  numerical color value;  numerical color value;  gastrointestinal tumor;  onco-life system;  narrow band imaging;  gastric cancer | h. pylori (10.49, 0.005);  numerical color value (ncv) (10.49, 0.005); diagnostic reliability (10.49, 0.005); fluorescence (10.49, 0.005);  white light endoscopy (10.49, 0.005) | early gastric cancer (0.09);  endoscopic submucosal |
| 24 | laparoscopic distal gastrectomy | 9 | 0.991 | 2016 | laparoscopic distal gastrectomy; laparoscopic gastrectomy;  intraoperative endoscopy;  endoscopic retroflexion;  gastric surgery;  resection margin;  gastric cancer;  preoperative endoscopic clipping; additional resection;  laparoscopic gastrectomy | laparoscopic distal gastrectomy  (22.48, 1.0E-4);  additional resection (11.14, 0.001);  gastric surgery (11.14, 0.001);  preoperative endoscopic clipping  (11.14, 0.001);  totally laparoscopic gastrectomy  (11.14, 0.001) | early gastric cancer (0.1);  endoscopic submucosal |
| 25 | serum opsonic activity | 6 | 0.999 | 2019 | physical stress;  endoscopic submucosal dissection; indirect calorimeter;  resting energy expenditure; chemiluminescence;  serum opsonic activity | serum opsonic activity (12.43, 0.001); chemiluminescence (12.43, 0.001);  physical stress (12.43, 0.001);  resting energy expenditure (12.43, 0.001); indirect calorimeter (12.43, 0.001) | early gastric cancer (0.1);  endoscopic submucosal |
